# 

**Published:** 1892-12

**Authors:** 


					CONTENTS.
The Teachings of Failure.
By E.
Markham Skerritt, 13.A., M.u.
Lond., F.R.C.P.
229
Two Cases of Locomotor Ataxy with
Charcot's Joint Disease.
By
Henry Davy, M.D. Lond., and
Arthur G. Blomfield, M.D.
Aberd
249
Health-Resorts in the West of
England and South Wales.
Bath.
By A. B. Brabazon, M.D.
The Earlv History of the Bristol
Medical School.
By Augustin
Prichard, F.R.C.S. Eng.
264 I
Progress of the Medical Sciences:
Medicine.
By Dr. J. E. Shaw
292
Surgery.
By Dr. James Swain ...
Obstetrics.
By L. M. Griffiths
303
Otology.
By W. H. Harsant
309
Reviews of Books
311
Notes on Preparations for the Sick
327
Meetings of Societies
329
The Library of the Bristol Medico-
Chirurgical Society   332
Scraps
339

				

